# The Congress of American Physicians and Surgeons

**Published:** 1889-11

**Authors:** 


THE CONGRESS OF AMERICAN PHYSICIANS AND SURGEONS.—Dr. C. H. Mastin, of Mobile, who may be said to be the father of this organization, set a wholesome example in the ground he took, at the recent meeting of the Executive Committee, for declining the nomination to the presidency. He is reported as having said that no member of the Executive Committee ought ever to be elected to the presidency. Thereupon Dr. S. Weir Mitchell, of Philadelphia, was chosen President, and Dr. William H. Carmalt, of New Haven, Secretary. The next meeting is to be held in Washington, in September, 1891.—*New York Medical Journal.*

